# Prevalence and Resistance Patterns of Pediatric Urinary Tract Infections in Bahrain

**DOI:** 10.7759/cureus.20859

**Published:** 2021-12-31

**Authors:** Omaima A Shaaban, Nermin A Mahmoud, Anas A Zeidan, Nitya Kumar, Alan C Finan

**Affiliations:** 1 Pediatrics, Royal College of Surgeons in Ireland - Bahrain, Busaiteen, BHR; 2 Epidemiology and Public Health, Royal College of Surgeons in Ireland - Bahrain, Busaiteen, BHR; 3 Pediatrics, King Hamad University Hospital, Busaiteen, BHR

**Keywords:** bahrain, uropathogenic escherichia coli, antibiotics resistance, paediatric, urinary tract infection

## Abstract

Background

Urinary tract infections (UTI) are a commonly encountered infection in the pediatric age group. Knowledge of the causative pathogens and their antimicrobial resistance patterns in specific geographical locations is important to provide optimum care. The aim of this study is to describe the prevalence and the antimicrobial resistance patterns of the pathogens causing UTI in the pediatric age group in one tertiary inpatient Pediatric unit in Bahrain.

Methods

This is a retrospective cross-sectional study, conducted at King Hamad University Hospital (KHUH), Bahrain. The inclusion criteria consisted of patients ≤ 14 years of age admitted to the Pediatrics department at KHUH with bacteriologically proven UTI between the months of January 2018 and May 2021. Patients who were identified to have chronic urinary tract conditions or neurodevelopmental problems involving the urinary tract were excluded from the study. Electronic medical records were used to collect data regarding the isolated pathogens and sensitivity testing results.

Results

A total of 242 cases with positive culture were included. The most common bacteria causing UTI in this sample were successively *Escherichia coli *(68.60%), *Klebsiella pneumoniae* (10.30%), *Proteus mirabilis* (4.69%) and *Pseudomonas aeruginosa* (3.31%) (p<0.01). *E. coli* was most resistant to cefazolin (94%), followed by ampicillin (62.68%), whilst it was most sensitive to nitrofurantoin (98.96%) followed by amikacin (98.43%) (p<0.01). *K. pneumoniae* showed the highest rate of resistance to ampicillin (95.24%) followed by cefazolin (83.33%), meanwhile having the highest sensitivity rate to amikacin (95.24%), followed by ciprofloxacin (90.48%). *P. mirabilis* had the highest resistance to cefazolin (100%) followed by nitrofurantoin (87.50%), while having the highest sensitivity to piperacillin/tazobactam (100%).

Conclusion

*E. coli* is the most common cause of UTI in the pediatric population and it was found to be most sensitive to nitrofurantoin and amikacin whilst being relatively resistant to cefazolin and ampicillin. Similarities between our study and previous studies around the world were found when comparing the antibiotics resistance patterns. Nevertheless, it is our recommendation that empirical antibiotic selection should be tailored to the local data collected from the region.

## Introduction

Urinary tract infections (UTIs) are a commonly encountered infection in the pediatric age group both in the community as well as in the hospital setting [1]. It is defined by the presence of a bacterial growth exceeding 10^5^ colony forming units per milliliter (CFU/ml) [2].

The predisposing factors leading to a UTI include the female gender, white ethnicity, history of prior UTI, dehydration, neurogenic bladder, diabetes mellitus, genitourinary instrumentation (e.g., indwelling urinary catheter, double J stent), congenital genitourinary malformation (e.g., vesicoureteral reflux, posterior urethral valves), phimosis, incomplete/infrequent voiding and chronic constipation [3,4].

Symptoms of UTI in infants and children up to two years of life include fever and occasionally symptoms of sepsis. They may also present with crying whilst voiding, change in urine color or a poor stream [5], whereas children older than two years of age with cystitis will present with urinary urgency, frequency, dysuria, enuresis, cloudy urine, malodorous urine, and suprapubic pain/ tenderness [6]. Children with pyelonephritis will experience urinary symptoms in addition to systemic involvement (fever, chills, and rigor) as well as flank pain and costovertebral angle tenderness [6].

As the clinical features can sometimes be non-specific in children, a midstream urine sample is required to confirm the diagnosis of a UTI [7]. The National Institute for Health and Care Excellence (NICE) guidelines recommend that the urine sample should preferably be collected by clean catch; however, if this was not possible, a urine collection pad can be utilized instead but not via cotton wool balls, gauze or sanitary towels. If non-invasive techniques are not feasible, a catheter sample or suprapubic aspirate under ultrasound guidance can be used [8].

Commonly known UTI-causing pathogens include* Escherichia coli* (accounting for approximately 85% of UTIs in children), *Klebsiella, Proteus, Enterobacter, Citrobacter, Staphylococcus saprophyticus* and *Enterococcus* [9]. Antibiotics commonly used to treat UTIs consist of ampicillin, nitrofurantoin, co-trimoxazole (trimethoprim/sulfamethoxazole) and ciprofloxacin [10]. Other choices include amoxicillin/clavulanate (Augmentin) or cephalosporins, such as cefixime (Suprax), cefpodoxime, cefprozil (Cefzil), or cephalexin (Keflex) [9].

The main objectives of management of childhood urinary tract infections are to achieve rapid recovery and prevent complications, such as urosepsis, urolithiasis, renal abscess and permanent renal parenchymal damage. To achieve this, empirical antibiotics are often prescribed even before the culture results are available. Antibiotic resistance of urinary tract pathogens is increasing worldwide, especially to commonly used antibiotics [11]. The increase in antibiotic resistance towards the Gram-negative Enterobacteriaceae family over the last two decades is highlighted by the emergence of extended spectrum beta-lactamase (ESBL) producing organisms [1].

This increase in antibiotic resistance is likely to have significant clinical implications for the empirical use of antibiotics. Knowledge of the causative pathogens of UTIs and their antimicrobial resistance patterns in specific geographical locations may aid physicians in choosing the appropriate antimicrobial empirical treatment [11]. Thus, the aim of this study is to provide information about these factors in our particular population of patients.

## Materials and methods

The aim of this retrospective cross-sectional study is to assess the prevalence of the pathogens isolated from the collected urine cultures in children admitted with microbiologically confirmed UTI during the duration starting from January 2018 and ending in May 2021; in addition to assessing the prevalence of antimicrobial resistance for these organisms. This study was conducted in the Pediatric department in King Hamad University Hospital (KHUH) which is a tertiary care hospital in the Kingdom of Bahrain.

The ICD-10 code for unspecified UTI was used in order to identify patients who have been diagnosed with a UTI in the electronic medical records. The electronic records of patients under this code were reviewed and patients with bacteriologically proven UTI were selected for inclusion in this study. Data on age, gender, urine culture results and sensitivity patterns for identified pathogens were obtained from the electronic medical records. All the recorded data were encrypted and no personal information about the patients was included.

In KHUH, the final diagnosis of UTI is confirmed based on clinical presentation and is further supported with the microbiology results. The collected urine sample is then sent to the microbiology department for analysis in addition to culture and sensitivity testing. A positive culture is considered if the urine sample showed bacterial growth >10^5^ colony-forming units of a significant pathogen. A manual colony count is done using a standardized loop (loop size of 1 microliter) as a tool for obtaining the standard urine quantity. Bruker MALDI-TOF was used for the identification of clinically relevant organisms and BD Phoenix M50 was used for both the identification of clinically relevant organisms as well as antibiotic sensitivity testing by measuring minimum inhibitory concentration (MIC). Common antibiotics used to treat UTI in the pediatric age group were included in this study. These antibiotics are ampicillin, nitrofurantoin, co-trimoxazole (trimethoprim/sulfamethoxazole), ciprofloxacin, co-amoxiclav (amoxicillin/clavulanate), cefazolin, gentamicin, amikacin, cefuroxime, ceftriaxone, piperacillin/tazobactam and ceftazidime.

Patients up to 14 years of age admitted to the pediatrics department with confirmed UTI were included whereas those with chronic urinary tract conditions and neurodevelopmental problems involving the urinary tract were excluded from the study.

Statistical Package for Social Sciences (SPSS) version 25.0 (IBM Corp., Armonk, NY, USA) was used for statistical analysis. Frequencies and percentages were computed for the categorical variables. Chi square tests were used to compute significant differences among the frequencies and p ≤0.05 was considered statistically significant. This study has been approved by the Institutional Review Board in King Hamad University Hospital, Bahrain (KHUH IRB) on 6 June 2021. 

## Results

Urine samples from 624 pediatric cases (<14 years old) were submitted for analysis and culture from January 2018 to June 2021 to confirm an initial clinical diagnosis of urinary tract infection. Sixty cases were excluded from the study due to the presence of chronic urinary tract conditions and neurodevelopmental problems involving the urinary tract. The included samples showed 242 cases (42.9%) with bacterial growth higher than 10^5^ CFU/ml (i.e., positive culture). The percentages of confirmed UTI among male and female cases suspected to have UTI were 40.76% (53/130) and 43.54% (189/434) respectively. Sixty-seven cases (27.6%) were included from 2018, 76 cases (31.4%) from 2019, 65 cases (27.2%) from 2020, and 34 cases (13.8%) from 2021.

The sample included 53 male cases (21.9%) and 189 female cases (78.10%) (p<0.01 between males and females). The included cases were categorized into three age groups: four cases (1.70%) were neonates (<28 days), 51 cases (21.10%) were infants (28 days - one year) and 186 cases (77.20%) were children (one year - 14 years) (p<0.01 between the age groups).

Twenty-five different bacteria were identified as the causative organism in our sample; the five most predominant agents were successively *E. coli* (68.60%), *Klebsiella pneumoniae* (10.30%), *Proteus mirabilis* (4.69%), *Pseudomonas aeruginosa* (3.31%), and *Staphylococcus saprophyticus* (2.10%) as shown in Table 1. 

**Table 1:** The most predominant agents causing urinary tract infections in the total sample, males and females. a: Chi-square test, b: percentage out of n = 242 (entire sample), c: percentage out of n = 53 (males), d: percentage out of n = 189 (females)

Bacteria	Number of cases	Percentage	P value between the organisms within each group
Total sample			
Escherichia coli	166	68.60%^b^	<0.01^a^
Klebsiella pneumoniae	25	10.30%^b^	
Proteus mirabilis	12	4.96%^b^	
Pseudomonas aeruginosa	8	3.31%^b^	
*Staphylococcus saprophyticus *(coagulase negative)	5	2.10%^b^	
Males			
Escherichia coli	19	35.85%^c^	<0.01^a^
Klebsiella pneumoniae	12	22.64%^c^	
Proteus mirabilis	6	11.32%^c^	
Pseudomonas aeruginosa	3	5.66%^c^	
Enterococcus faecalis	3	5.66%^c^	
Females			
Escherichia coli	147	77.77%^d^	<0.01^a^
Klebsiella pneumoniae	13	6.88%^d^	
Proteus mirabilis	6	3.17%^d^	
Pseudomonas aeruginosa	5	2.65%^d^	
*Staphylococcus saprophyticus *(coagulase negative)	3	1.58%^d^	

The results revealed that *E. coli* occurred more frequently in females compared to males (77.77% in females vs. 35.85% in males, p<0.01). The results showed that although the frequencies of the other three predominant organisms (*K. pneumoniae, P. mirabilis, *and* P. aeruginosa*) were higher in males, there was no significant difference between males and females (p value between males and females = 0.84, 1.00, and 0.48 for *K. pneumoniae, P. mirabilis, and P. aeruginosa* respectively).

*E. coli* and *K. pneumoniae* successively were found to be the most predominant organisms causing UTI in all the three age groups (Table 2). The frequency of *E. coli* was the highest in infants (75%) followed by children (69.52%) and then by neonates (64.71%) with a p value <0.01 between the three age groups. The frequency of *K. pneumoniae* was highest in infants (25%) followed by neonates (15.69%) followed by children (8.56%) with p value <0.001 between the three age groups.

**Table 2:** The most predominant agents causing urinary tract infections in each age group. a: Chi-square test, b: percentage out of n=4 (neonates), c: percentage out of n=51 (infants), d: percentage out of n=187 (children)

Organism	Number of cases	Percentage	P value between the organisms within each group
Neonates			
Escherichia coli	3	75%^b^	0.56^a^
Klebsiella pneumoniae	1	25%^b^	
Proteus mirabilis	0	0%^b^	
Pseudomonas aeruginosa	0	0%^b^	
*Staphylococcus saprophyticus *(coagulase negative)	0	0%^b^	
Infants			
Escherichia coli	33	64.71%^c^	<0.01^a^
Klebsiella pneumoniae	8	15.69%^c^	
Pseudomonas aeruginosa	4	7.84%^c^	
Stenotrophomonas maltophilia	2	3.92%^c^	
Proteus mirabilis	1	1.96%^c^	
Children			
Escherichia coli	130	69.52%^d^	<0.01^a^
Klebsiella pneumoniae	16	8.56%^d^	
Proteus mirabilis	11	5.88%^d^	
*Staphylococcus saprophyticus *(coagulase negative)	5	2.67%^d^	
Pseudomonas aeruginosa	4	2.14%^d^	

Out of the 166 cases of UTI caused by *E. coli*, 39 cases (23.49%) were of ESBL-producing strain. A statistically significant difference was found between males and females in the percentage of ESBL-producing *E. coli* (42.10% for males vs 21.00% for females, p<0.01) in addition to a statistically significant difference between the three age groups (36.36% for neonates, 0% for infants, and 20.70% for children, p=0.016).

**Figure 1:**
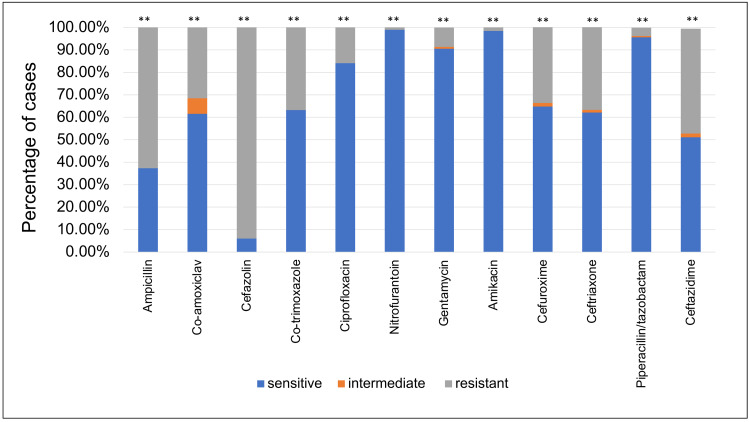
E. coli sensitivity patterns to the antibiotics used to treat urinary tract infections. This bar chart shows the sensitivity of each antibiotic (as a percentage of cases) when tested against* E. coli* samples, **: p<0.01

*E. coli *displayed the highest resistance to cefazolin (94%), followed by ampicillin (62.68%) and it showed the highest sensitivity rate to nitrofurantoin (98.96%) followed by amikacin (98.43%) (Figure 1).

**Figure 2:**
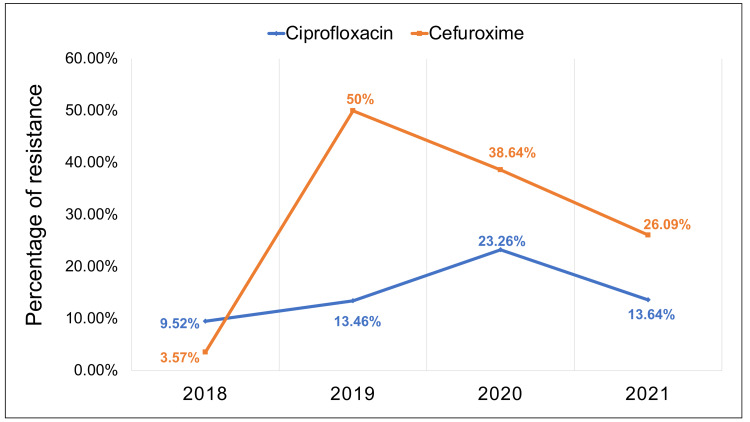
E. coli resistance to ciprofloxacin and cefuroxime over the study period. This line chart shows the change of E. coli resistance to the antibiotics ciprofloxacin and cefuroxime over the study period (2018-2021). The resistance of the two antibiotics changed significantly over the period of the study (2018-2021) with p value = 0.015 and p value = 0.008 for ciprofloxacin and cefuroxime respectively.

*E. coli’s* resistance to ciprofloxacin and cefuroxime changed significantly over the period of this study (p<0.015 and p<0.008 for ciprofloxacin and cefuroxime respectively) (Figure 2).

**Figure 3:**
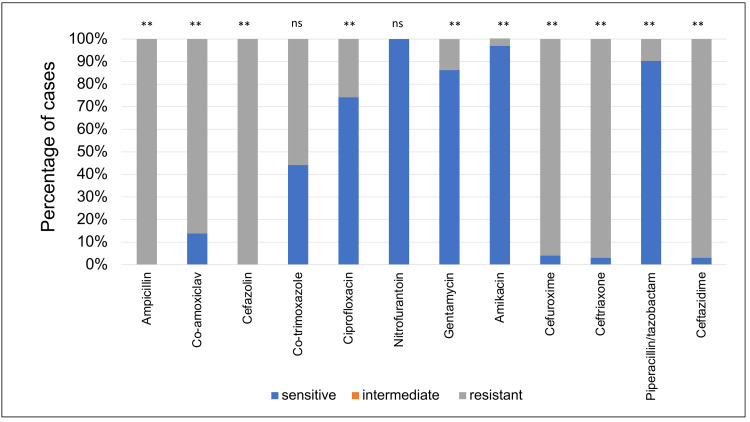
Extended spectrum beta-lactamase (ESBL)-producing E. coli sensitivity patterns to the antibiotics used to treat urinary tract infections. This bar chart shows the sensitivity of each antibiotic (as a percentage of cases) when tested against ESBL-producing E. coli samples, **: p<0.01, ns: p>0.05

The ESBL-producing *E. coli *strain showed the highest resistance to ampicillin (100%) and cefazolin (100%) but the highest sensitivity to nitrofurantoin (100%) followed by amikacin (96.97%) (Figure 3).

**Figure 4:**
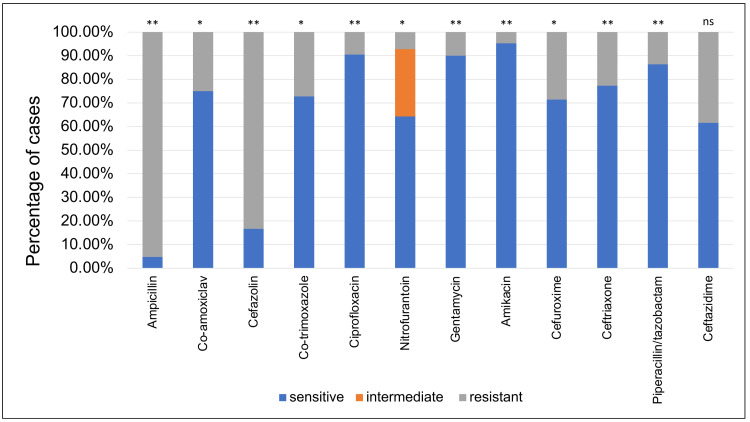
Klebsiella pneumoniae sensitivity patterns to the antibiotics used to treat urinary tract infections. This bar chart shows the sensitivity of each antibiotic (as a percentage of cases) when tested against *Klebsiella pneumoniae* samples, **: p<0.01, *: p<0.05, ns: p>0.05

*K. pneumoniae* showed the highest resistance to ampicillin (95.24%) then by cefazolin (83.33%) whilst having the highest sensitivity rate to amikacin (95.24%), followed by ciprofloxacin (90.48%) (Figure 4).

*P. mirabilis* had the highest resistance to cefazolin (100%) followed by nitrofurantoin (87.50%) meanwhile having the highest sensitivity towards piperacillin/tazobactam (100%) followed by co-trimoxazole (83.33%). *P. aeruginosa* had a resistance rate of 100% to five antibiotics (ampicillin, co-amoxiclav, cefazolin, cefuroxime and ceftriaxone) and sensitivity of 100% to four antibiotics (ciprofloxacin, gentamicin, amikacin and ceftazidime). Table 3 shows the percentage of resistance of the most predominant organisms in this study as well as for those of the antibiotics overall regardless of the organism. Table 3 also shows the number of cases in which the resistance of an antibiotic was not tested in each organism as well as in the overall sample.

**Table 3:** Antibiotic resistance rate in the most predominant organisms causing urinary tract infections. RR: Resistance rate [percentage of resistant cases/total number of cases (excluding the number of cases in which the test was not done)], a: “Not done” is the number of cases in which the resistance of this antibiotic was not tested.

	Overall (N=242)		*Escherichia coli *(n=166)		ESBL-producing* E. coli * (n=39)		*Klebsiella pneumoniae *(n=25)		*Proteus mirabilis *(n=12)		*Pseudomonas aeruginosa *(n=8)	
	RR	Not done ^a^	RR	Not done ^a^	RR	Not done ^a^	RR	Not done^a^	RR	Not done ^a^	RR	Not done^a^
Ampicillin	67.50%	42/242	62.68%	24/166	100%	3/39	95.24%	4/25	55.56%	3/12	100%	3/8
Co-amoxiclav	34.97%	59/242	31.54%	36/166	86.20%	10/39	25%	5/25	20%	2/12	100%	2/8
Cefazolin	94.12%	174/242	94%	116/166	100%	10/39	83.33%	19/25	100%	10/12	100%	3/8
Co-trimoxazole	33.01%	36/242	36.81%	22/166	55.88%	5/39	27.27%	3/25	16.67%	0/12	83.33%	2/8
Ciprofloxacin	13%	42/242	15.94%	28/166	25.80%	8/39	9.52%	4/25	22.22%	3/12	0%	0/8
Nitrofurantoin	11.27%	100/242	1.04%	70/166	0%	16/39	7.14%	11/25	87.50%	4/12	66.67%	2/8
Gentamycin	10.38%	59/242	8.66%	39/166	13.70%	10/39	10%	5/25	22.22%	3/12	0%	2/8
Amikacin	4%	67/242	1.57%	39/166	3.30%	6/39	4.76%	4/25	28.57%	5/12	0%	1/8
Cefuroxime	36.60%	48/242	33.79%	21/166	96.00%	14/39	28.57%	4/25	25%	0/12	100%	2/8
Ceftriaxone	35.68%	57/242	37.12%	34/166	96.97%	6/39	22.73%	3/25	33.33%	3/12	100%	2/8
Piperacillin/Tazobactam	5.79%	52/242	3.68%	30/166	9.67%	8/39	13.64%	3/25	0%	3/12	25%	0/8
Ceftazidime	42.86%	116/242	46.67%	76/166	96.97%	6/39	38.46%	11/25	40%	7/12	0%	0/8

## Discussion

Urinary tract infection (UTI) is a commonly encountered infection in the pediatric age group with high clinical importance due to its high morbidity and mortality rates [1,12]. Antibiotic resistance of UTI-causing organisms is increasing worldwide and knowledge regarding the causative pathogens and their antimicrobial resistance patterns in the local setting is essential to provide a more tailored and effective treatment [11]. The aim of this study is to provide information about these factors in the Kingdom of Bahrain.

In this study, urine samples from 564 pediatric cases (14 years old) were submitted for urine analysis and culture to confirm an initial clinical diagnosis of a urinary tract infection. Out of these cases, 242 cases (42.9%) were confirmed to have a UTI (i.e. positive culture). In our study, the percentage of confirmed UTI among male and female cases suspected to have UTI was 40.76% and 43.54% respectively (male:female ratio 1:1.07); Mirsoleymani et al. found this ratio to be 1:4, Farajnia et al. found it to be 1:2 and finally Farrell et al. found it to be 1:1.41 [11,13,14].

In this study, the number of female cases (78.10%) was found to be significantly higher (p<0.01) in comparison to the male cases (21.90%). This is similar to other studies which found the percentage of female pediatric cases to be higher than the male cases ranging from 66.8% to 86% [11,15-18]. This could be due to the fact that females have shorter urethra [7]. The prevalence of UTI is higher in males than females in the first three months of life, however after the first year of life it is higher in females [11,19]. This can be explained by the higher incidence of structural anomalies among males in the first three months of life [11]. Since 77.20% of our sample are children between the ages of one year and 14 years, this explains the high prevalence among females in our study. A study with higher percentage of neonates and infants (<one year old) such as the one done by Mirsoleymani et al. (percentage of neonates and infants combined is 65.2%) had a higher percentage of male cases (55.9%) which supports our proposed explanation [11].

*E. coli* was the most common pathogen causing UTI in our sample, accounting for 68.60% of all urinary isolates. This is similar to previous studies in which* E. coli* was found to be the most predominant UTI-causing organism ranging from 65.2% to 87% of all the UTI cases in a pediatric sample [11,15-17,19-24]. The second most common organism causing UTI was *K. pneumoniae* (10.30%) followed by *P. mirabilis* (4.96%) and *P. aeruginosa* (3.31%) with a p value <0.01 between the groups. In a similar study done on a pediatric sample of 361 patients in the United States, *K. pneumoniae* was also found to be the second most common organism causing UTIs (3%) [16]; this is further supported by another study which was conducted in Iran with *Klebsiella* causing 36.2% of UTIs in the pediatric age group [11]. On the other hand, a study done in Turkey found that *Proteus* is the second most common organism (19.0%) causing UTI in the pediatrics age group followed by *Klebsiella* (14.7%) [15].

The four most predominant organisms that result in a UTI in the overall sample were also the most predominant organisms causing UTI in both males and females. The prevalence of *E. coli *was significantly higher among females (77.77%) than males (35.85%) (p<0.01). This was similar to other studies which found *E. coli *prevalence to be higher in females (ranging from 70.8% to 83%) compared to males (ranging from 50% to 60.5%) with p value <0.05 in all the compared studies [11,15,17,21]. There was no significant difference between males and females (p>0.05) in the prevalence of *K. pneumoniae, P. mirabilis* and *P. aeruginosa*. This, however, was contradictory to other reviewed literatures which found the prevalence of the less common organisms (i.e. organisms that are not *E. coli*) to be significantly higher in males. For example, Mirsoleymani et al. reported that the prevalence of UTI due to *Klebsiella* spp. as well as *P. aeruginosa* were higher in males than in females (p=0.001 and p=0.035 respectively) [11]. One study done in the United States found that every species causing UTI in the pediatric sample other than *E. coli* was more common in males than females (p<0.001), with *Enterococcus* (17%), *Proteus* (11%) and *Klebsiella* (10%) being the next most common isolates in males after* E. coli,* meanwhile in females, these organisms each only accounted for ≤5% of the urinary isolates (p<0.001) [17]. *E. coli* was the most common organism causing UTI in the three age groups (p<0.01), followed by *K. pneumonia *(p<0.01). The prevalence of *E. coli *was the highest in infants (75%) followed by children (69.52%) and finally by neonates (64.71%) (p<0.01). Mirsoleymani et al. reported similar results of *E. coli* prevalence being the highest in infants (63.1%, p<0.001) [11]. Overall, in line with the other studies, our results indicate that there are significant differences in the organisms causing UTI in a pediatrics sample based on both age and gender and thus this should be taken into consideration while prescribing the empirical antibiotics [17,19].

According to our results, *E. coli *demonstrated the highest rate of resistance to cefazolin (94%, p<0.01), followed by ampicillin (62.68%, p<0.01) and then ceftazidime (46.67%, p<0.01). Several prior studies carried out on urine samples collected from children with confirmed UTIs have reported *E. coli* to have the highest resistance to ampicillin with the percentage of resistance ranging from 45% to 83.5% and a p value <0.05 in all the compared studies [11,15-17]. Furthermore, the percentage of *E. coli *resistance to co-trimoxazole was found to be 36.81% (p value = 0.002). Other literature stated co-trimoxazole to be one of the most resisted antibiotics by *E. coli* with the percentage of resistance ranging from 24% to 75.4%, p value <0.05, in all the included studies [11,15-17]. Additionally, *E. coli *has also been found to have the highest sensitivity rate to nitrofurantoin (98.96%, p<0.01), followed by amikacin (98.43%, p<0.01). This was similar to other studies that demonstrated *E. coli* to be highly sensitive to nitrofurantoin ranging from 90.5% to >99% [11,15-17,21, 23]. Amikacin (the antibiotic with the second-highest sensitivity percentage in our study) has been found to be the most sensitive antibiotic in treating UTI caused by *E. coli *in several studies with sensitivity ranging from 79.7% to 100% [11,15-17].

The percentage of *E. coli* resistance to ciprofloxacin has increased significantly over the period of this study from 9.52% in 2018 to 23.26% in 2020 (p=0.015). A study done in the United States noted a similar pattern in ciprofloxacin resistance which increased 10-fold between 2004 and 2009 (males: 10% versus 1% and females: 4% versus 0.6%) [17]. Another study done in Turkey reported a similar pattern in which *E. coli* resistance to ciprofloxacin increased from 6.6% in 2009 to reach 14.7% in 2014 (p value <0.001) [15]. Several studies have demonstrated this pattern of increase in ciprofloxacin resistance over the years, one of the proposed explanations was that although ciprofloxacin is infrequently prescribed for pediatric UTIs, the use of this antibiotic has increased in adults over the last decade which might be the reason behind these changes [25-27].

Extended spectrum beta-lactamases (ESBLs) are class A β-lactamases that hydrolyse penicillin, oxyimino-cephalosporins and monobactams [28]. A study done in Turkey found that 64.5% of pediatric UTIs caused by *E. coli* were ESBL positive [29]. In our study, ESBL contributed to 23.49% of the total number of patients with *E. coli*. 42.1% of males with *E. coli *were found to be ESBLs (p<0.01) compared to 21% of the females (p<0.01). The highest resistance for ESBLs were for ampicillin and cefazolin (100% for each of the antibiotics) with a p<0.01 in both. Nitrofurantoin had the highest sensitivity rates (100%, p<0.01) followed by amikacin (96.97%, p<0.01).

In our study,* K. pneumoniae* (the second most common organism isolated) had the highest rate of resistance to ampicillin (95.24%, p<0.01), and the lowest resistance to amikacin (4.76%, p<0.01). These results were similar to other studies which found *Klebsiella* isolates to have the highest resistance to ampicillin ranging from 80.7% to 86.3% [11,15,17], and the least resistance to amikacin ranging from 2.4% to 26.9% [11,15].

*P. mirabilis* (the third most common organism isolated in our sample) was found to have the highest resistance to cefazolin (100%) followed by nitrofurantoin (87.50%, p=0.03), but the lowest resistance to piperacillin/tazobactam (0%) followed by co-trimoxazole (83.33%, p=0.021). Previous literature done on pediatric samples has reported *Proteus* spp. to have high resistance to nitrofurantoin (72.6% and 94%) [15,17], however, the resistance level of ampicillin and co-trimoxazole varied between the studies. *Proteus* isolates were noted to have low resistance in response to co-trimoxazole (11%) by a study done in the United States whereas it had a high rate of resistance (72.6%) in another study done in Turkey [15,17].

A major limitation of this study is its retrospective nature, this limits the study design to the variables that are present in the electronic medical records. For instance, the resistance patterns of some antibiotics were not measured for some isolates and were not included in the electronic medical records (Table 3).

## Conclusions

In conclusion, as per the conducted study, the most common pathogen identified in causing a UTI in patients ≤14 years of age is *E. coli*. This microorganism was found to be sensitive to both nitrofurantoin and amikacin whilst being resistant to cefazolin and ampicillin. Similarities between our studies and previous studies done around the world were found when comparing the antibiotic resistance patterns. Nevertheless, we recommend that empirical antibiotic selection should be based on the local data collated regarding resistance and sensitivity patterns observed in that region.

## References

[1] Hanna-Wakim RH, Ghanem ST, El Helou MW (2015). Epidemiology and characteristics of urinary tract infections in children and adolescents. Front Cell Infect Microbiol.

[2] Larcombe J (1999). Urinary tract infection in children. BMJ.

[3] Simon C, Everitt H, Van Dorp F, Hussain N, Nash E, Peet D (2020). Oxford Handbook of General Practice.

[4] Gondim R, Azevedo R, Braga AA, Veiga ML, Barroso U Jr (2018). Risk factors for urinary tract infection in children with urinary urgency. Int Braz J Urol.

[5] Vasudev AS, Shah NK (2017). Algorithms in Pediatrics. https://prithvibooks.com/product/algorithms-in-pediatrics-by-anand-s-vasudev/.

[6] Leung AK, Wong AH, Leung AA, Hon KL (2019). Urinary tract infection in children. Recent Pat Inflamm Allergy Drug Discov.

[7] Kaufman J, Temple-Smith M, Sanci L (2019). Urinary tract infections in children: an overview of diagnosis and management. BMJ Paediatr Open.

[8] Mori R, Lakhanpaul M, Verrier-Jones K (2007). Diagnosis and management of urinary tract infection in children: summary of NICE guidance. BMJ.

[9] White B (2011). Diagnosis and treatment of urinary tract infections in children. Am Fam Physician.

[10] Alanazi MQ, Alqahtani FY, Aleanizy FS (2018). An evaluation of E. coli in urinary tract infection in emergency department at KAMC in Riyadh, Saudi Arabia: retrospective study. Ann Clin Microbiol Antimicrob.

[11] Mirsoleymani SR, Salimi M, Shareghi Brojeni M, Ranjbar M, Mehtarpoor M (2014). Bacterial pathogens and antimicrobial resistance patterns in pediatric urinary tract infections: a four-year surveillance study (2009-2012). Int J Pediatr.

[12] Mortazavi F, Shahin N (2009). Changing patterns in sensitivity of bacterial uropathogens to antibiotics in children. Pak J Med Sci.

[13] Farajnia S, Alikhani MY, Ghotaslou R, Naghili B, Nakhlband A (2009). Causative agents and antimicrobial susceptibilities of urinary tract infections in the northwest of Iran. Int J Infect Dis.

[14] Farrell DJ, Morrissey I, De Rubeis D, Robbins M, Felmingham D (2003). A UK multicentre study of the antimicrobial susceptibility of bacterial pathogens causing urinary tract infection. J Infect.

[15] Erol B, Culpan M, Caskurlu H (2018). Changes in antimicrobial resistance and demographics of UTIs in pediatric patients in a single institution over a 6-year period. J Pediatr Urol.

[16] Lutter SA, Currie ML, Mitz LB, Greenbaum LA (2005). Antibiotic resistance patterns in children hospitalized for urinary tract infections. Arch Pediatr Adolesc Med.

[17] Edlin RS, Shapiro DJ, Hersh AL, Copp HL (2013). Antibiotic resistance patterns of outpatient pediatric urinary tract infections. J Urol.

[18] Badhan R, Singh DV, Badhan LR, Kaur A (2016). Evaluation of bacteriological profile and antibiotic sensitivity patterns in children with urinary tract infection: a prospective study from a tertiary care center. Indian J Urol.

[19] Afsharpaiman S, Bairaghdar F, Torkaman M, Kavehmanesh Z, Amirsalari S, Moradi M, Safavimirmahalleh M (2012). Bacterial pathogens and resistance patterns in children with community-acquired urinary tract infection: a cross sectional study. J Compr Ped.

[20] Panahi Y, Beiraghdar F, Moharamzad Y, Matinzadeh ZK, Einollahi B (2008). The incidence of urinary tract infections in febrile children during a two-year period in Tehran, Iran. Trop Doct.

[21] Prais D, Straussberg R, Avitzur Y, Nussinovitch M, Harel L, Amir J (2003). Bacterial susceptibility to oral antibiotics in community acquired urinary tract infection. Arch Dis Child.

[22] Ashkenazi S, Even-Tov S, Samra Z, Dinari G (1991). Uropathogens of various childhood populations and their antibiotic susceptibility. Pediatr Infect Dis J.

[23] Goldraich NP, Manfroi A (2002). Febrile urinary tract infection: Escherichia coli susceptibility to oral antimicrobials. Pediatr Nephrol.

[24] Al Sweih N, Jamal W, Rotimi VO (2005). Spectrum and antibiotic resistance of uropathogens isolated from hospital and community patients with urinary tract infections in two large hospitals in Kuwait. Med Princ Pract.

[25] Copp HL, Shapiro DJ, Hersh AL (2011). National ambulatory antibiotic prescribing patterns for pediatric urinary tract infection, 1998-2007. Pediatrics.

[26] Caterino JM, Weed SG, Espinola JA, Camargo CA Jr (2009). National trends in emergency department antibiotic prescribing for elders with urinary tract infection, 1996-2005. Acad Emerg Med.

[27] Kahlmeter G (2000). The ECO.SENS Project: a prospective, multinational, multicentre epidemiological survey of the prevalence and antimicrobial susceptibility of urinary tract pathogens--interim report. J Antimicrob Chemother.

[28] Shakya P, Shrestha D, Maharjan E, Sharma VK, Paudyal R (2017). Extended-spectrum βlactamase producing among Escherichia coli and Klebsiella spp causing urinary tract infections; a hospital based study. Open Microbiol J.

[29] Topaloglu R, Er I, Dogan BG (2010). Risk factors in community-acquired urinary tract infections caused by ESBL-producing bacteria in children. Pediatr Nephrol.

